# An asymptomatic double-chambered left ventricle diagnosed by contrast-enhanced ultrasound imaging: A case report

**DOI:** 10.1097/MD.0000000000033524

**Published:** 2023-04-21

**Authors:** Jie Tian, Dan Jin, Ying Zhu, Yani Liu

**Affiliations:** a Department of Medical Ultrasound, Tongji Hospital, Tongji Medical College, Huazhong University of Science and Technology, Wuhan, China.

**Keywords:** congenital heart disease, contrast enhance-ultrasound imaging, double-chambered left ventricle, echocardiography

## Abstract

The double-chambered left ventricle (DCLV) is a rare congenital heart disease and is separated into 2 chambers by abnormally hypertrophied bundles of muscle or fibrous strips. Differential diagnoses, especially diverticulum, aneurysms and large ventricular septal defect are sometimes difficult.

**Patient concerns:**

A 33-year-old woman was admitted to the hospital to undergo abortion without any discomfort such as palpitation, chest tightness, shortness of breath and etc. The electrocardiogram reported a suspicious left anterior branch block and extensive anterior wall R-wave incremental dysplasia. The transthoracic echocardiography showed a bilayer structure of the ventricular septum with a continuity interruption visible on the left ventricular surface, and the color doppler flow imaging showed a low velocity bidirectional flow at this continuity interruption, communicated with the left ventricular cavity.

**Diagnoses:**

Final diagnosis of DCLV was confirmed by contrast-enhance ultrasound imaging.

**Interventions:**

The patient was discharged without any special treatment of the heart after the abortion.

**Outcomes:**

The patient did not complain of any special discomfort after the 3, 6, and 9 months of outpatient follow-ups.

**Lessons:**

This case highlights the necessity of contrast-enhance ultrasound imaging, which plays an important role in improving the accuracy of DCLV diagnosis and in differentiating it from other diseases.

## 1. Introduction

The double-chambered ventricle is a rare congenital heart disease, which the ventricle is separated into 2 chambers by abnormally hypertrophied bundles of muscle or fibrous strips. The double-chambered left ventricle (DCLV) is an extremely rare anomaly with few cases had been previously reported compared to double-chambered right ventricle.^[1]^ The patients are often asymptomatic. Echocardiography is an important imaging method in the diagnosis of this disease. We reported a case of DCLV unintentionally discovered by echocardiography. We also examined the value of contrast-enhance ultrasound (CEUS) imaging in the left heart for diagnosing DCLV.

## 2. Case presentation

**Figure 1.**
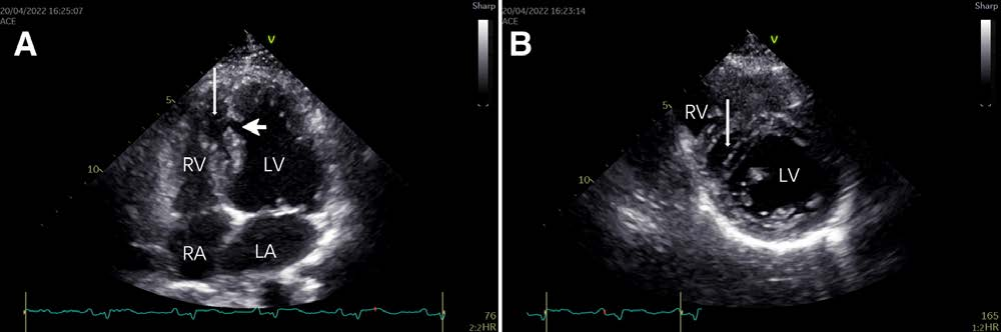
Two-dimensional (2D) echocardiography. (A) The apical 4-chamber view showed the bilayer structure of the ventricular septum (long arrow), and the continuity interruption in the left ventricular surface (short arrow). (B) The parasternal papillary muscle short-axis view clearly showed the bilayer structure of the ventricular septum (long arrow). LA = left atrium, LV = left ventricle, RA = right atrium, RV = right ventricle.

**Figure 2.**
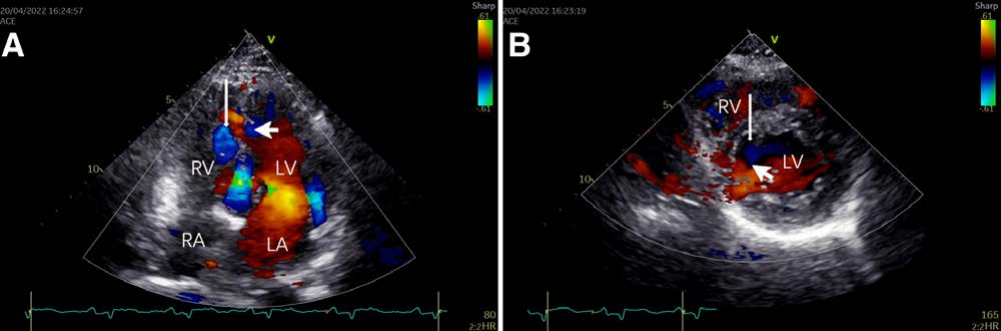
Color doppler flow imaging (CDFI). The apical 4-chamber view, (A) and parasternal papillary muscle short-axis view, (B) with CDFI revealed low velocity bidirectional flow at the continuity interruption (short arrows). The bidirectional flow communicated the bilayer structure of the ventricular septum (long arrows) with the left ventricular cavity.

**Figure 3.**
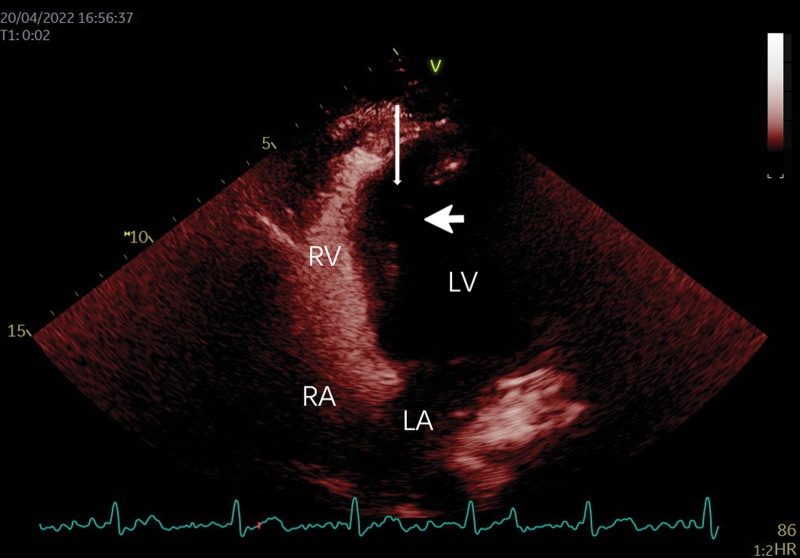
Contrast enhance-ultrasound (CEUS) imaging. The apical 4-chamber view showed the right atrium and right ventricle were sequentially visualized, no contrast was visualized in the bilayer structure of the ventricular septum (long arrows) or in the left ventricular cavity and the continuity interruption (short arrows). There were no negative contrast areas in the right ventricular cavity.

**Figure 4.**
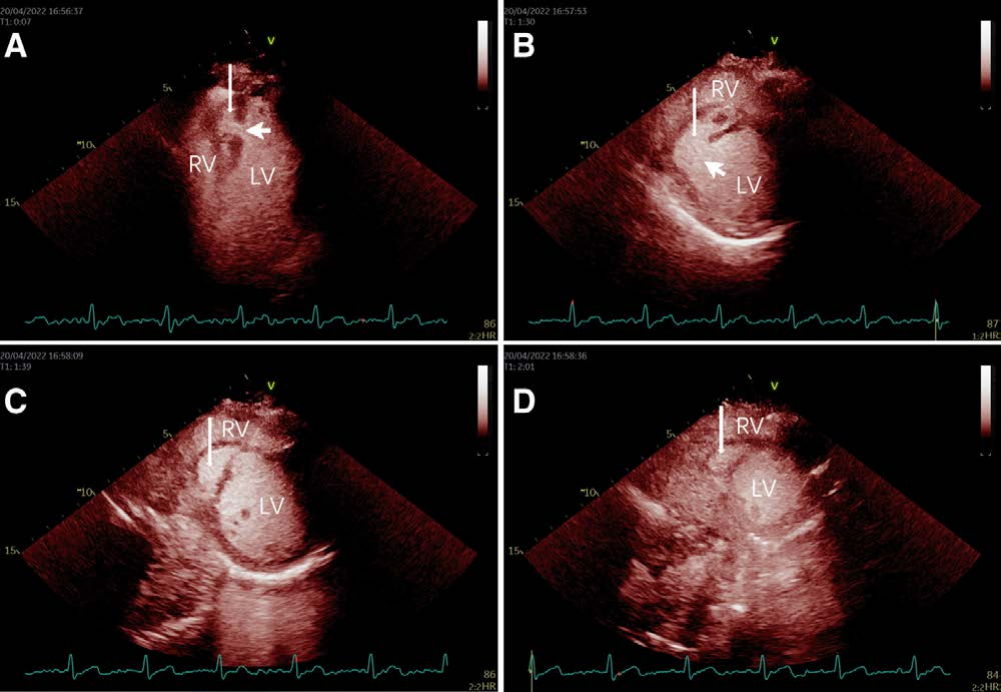
Contrast enhance-ultrasound (CEUS) imaging. The apical 4-chamber view, (A) the parasternal left ventricle short-axis view, (B) the parasternal papillary muscle short-axis view, (C) and the parasternal apical short-axis view, and (D) showed sequential visualization of the left atrium and left ventricle, the contrast agent was seen to enter the bilayer structure (long arrows) from the left ventricular cavity through the continuity interruption (short arrows) and was visualized simultaneously with the left ventricle.

The patient was a 33-year-old woman. In April 2022, she was admitted to the hospital for “2 + months of menopause and 2 days of embryonic arrest” to undergo abortion without any discomfort such as palpitation, chest tightness, shortness of breath and etc. She had no related histoire familiale. Physical examination and relevant laboratory tests did not show any significant abnormalities. The suspicious left anterior branch block and extensive anterior wall R-wave incremental dysplasia were found in electrocardiogram, then the transthoracic echocardiography (TTE) was suggested. The TTE showed a bilayer structure of the ventricular septum with a continuity interruption visible on the left ventricular surface (Fig. 1). The Color doppler flow imaging showed a low velocity bidirectional flow at this continuity interruption, which communicated with the left ventricular cavity (Fig. 2). The CEUS imaging was then conducted by injecting 1 ml Sono Vue (Bracco, Italy) contrast through the left upper limb elbow vein. The CEUS imaging showed that the right atrium and right ventricle were sequentially visualized, and during this time, no contrast was visualized in the septal bilayer structure or in the left ventricular cavity. There were also no negative contrast areas in the right ventricular cavity (Fig. 3). Subsequently, the left atrium and left ventricle were sequentially visualized, and the contrast agent was seen to enter the bilayer structure from the left ventricular cavity through the continuity interruption and was visualized simultaneously with the left ventricle (Fig. 4). The septal bilayer structure is actually part of the left ventricular cavity, which is a double-chambered left ventricle. The patient did not complain of any special discomfort after the abortion and was discharged without any special treatment of the heart. After the 3, 6, and 9 months of outpatient follow-ups by the TTE, nothing has changed in the heart and the patient was in good health without any special discomfort yet.

## 3. Discussion

The double-chambered ventricle is a rare congenital heart disease that is caused by abnormal myocardial development. The double-chambered ventricle is classified into right and left ventricular double-chambered heart, of which left ventricular double-chambered heart is more rarely.^[1]^ A DCLV refers to an abnormally hypertrophied bundles of muscle or fibrous strips separating the left ventricle into 2 chambers, named the main chamber and the accessory chamber. Usually, the main chamber is located at the proximal base, and the mitral and aortic valve orifices are often within the main chamber. The accessory chamber is located in the distal apical or lateral wall. Based on the location of the main and accessory chambers, the DCLV has 2 types, type A (upper and lower arrangement) and type B (left and right arrangement), and most of the types of DCLV reported in the literature are mostly type A.^[2–4]^ The symptoms of DCLV are related to the obstruction between the 2 chambers and the function of the left ventricle, and also related to the combination with other congenital heart diseases, such as atrial septal defect and tetralogy of Fallot. The clinical manifestations include palpitations, chest tightness after exertion, shortness of breath, dyspnea, cough, hemoptysis, cardiac murmur, arrhythmia and even left heart failure and pulmonary edema.^[5]^ In this case, the patient was an asymptomatic solitary B-type. The left ventricle was separated by an abnormally hypertrophied bundle of muscle into 2 chambers, with the main chamber on the left and the accessory chamber parallel on the right. The main chamber was located at the base and connected to the mitral and aortic valves, in which the size and contractile function were normal. The accessory chamber was located at the lateral, communicated with the main chamber through a continuity interruption, the wall motion and hemodynamics were unaffected. There was no evident of other anomalies during the examination, therefore, the patient was asymptomatic for years.

TTE is a noninvasive and easy imaging method to perform. Multifaceted examinations not only reveal the origin and orientation of the abnormal muscle bundles, the size, position, wall thickness, wall motion, but also evaluate the communication between the 2 chambers and determine whether there is obstruction between them.^[6]^ Expect for the above widespread applications, the CEUS imaging can improve the imaging of the left ventricular membrane, especially of the endometrium. It can clearly show the apical structure, enhance the resolution of the intraventricular border, and visualize the morphology and structure of the main and accessory chambers. The CEUS imaging can also assess the presence of other congenital heart diseases and be used to observe left ventricular myocardial blood perfusion for evaluating the coronary microcirculation.^[7]^ It is also useful for differential diagnoses.

The DCLV needs to be differentiated from diverticula, aneurysms, and large ventricular septal defect. The diverticulum is a rare congenital heart disease, manifested by dilated tumor cavity communicating with the left ventricular cavity through a small caliber tumor neck. It is mostly located in the apical and inferior posterior wall, not separated by abnormal tissue. The CEUS imaging shows a synchronous imaging of the tumor walled myocardium with the surrounding normal myocardium, and there is no contrast filling defect. The aneurysms, here mainly refer to the true aneurysm, mostly a complication of myocardial infarction, manifested by localized thinning of the left ventricular wall, saccular protrusion, echogenic enhancement, loss of systolic motion amplitude or paradoxical motion. It usually has a wider tumor neck. The CEUS imaging shows sparse and uneven contrast perfusion in the tumor wall. The large ventricular septal defect appears as an interruption of septal continuity and left-to-right shunt. When combined with severe pulmonary hypertension it can manifest as a bidirectional shunt. The CEUS imaging shows negative contrast areas in the right ventricle. In our case, the CEUS imaging showed that the left ventricular myocardium was well perfused, with which is helpful for distinguishing the DCLV from the true aneurysm. The CEUS imaging showed the contrast agent entered the bilayer structure from the left ventricular cavity through the continuity interruption and was visualized simultaneously with the left ventricle, and there were no negative contrast areas in the right ventricular cavity, which is helpful for distinguishing the DCLV from ventricular septal defect. Both TTE and CEUS imaging can clearly visualize the abnormal bundles of muscle, which is easily distinguished with diverticulum.

The treatment depends on the function of the main left ventricle and the combined malformations. When the DCLV occurs with obvious clinical manifestations and other cardiac malformations requiring correction, the surgical treatment is required. If no obvious hemodynamic abnormality is identified, a regular follow-up can be performed.^[8]^ As in the present case, the patient did not complain of any special discomfort, she was discharged without any special treatment of the heart. After 9 months of outpatient follow-ups, the patient was still in good health without any special discomfort.

In conclusion, the DCLV is a rare congenital heart malformation to diagnosis. The TTE is the primary method for the diagnosis of DCLV. The CEUS imaging plays an important role in improving the accuracy of DCLV diagnosis and has an irreplaceable value for the differential diagnoses. These applications with CEUS imaging are important in the clinical diagnosis, treatment, and assessment of prognosis for DCLV.

## Author contributions

**Conceptualization:** Yani Liu.

**Data curation:** Jie Tian.

**Investigation:** Dan Jin, Ying Zhu.

**Methodology:** Ying Zhu, Yani Liu.

**Validation:** Ying Zhu.

**Visualization:** Yani Liu.

**Writing – original draft:** Jie Tian.

**Writing – review & editing:** Yani Liu.
